# Predicting Depression Severity from Spontaneous Speech as Prompted by a Virtual Agent

**DOI:** 10.1192/j.eurpsy.2023.387

**Published:** 2023-07-19

**Authors:** A. König, M. Mina, S. Schäfer, N. Linz, J. Tröger

**Affiliations:** ^1^Cobtek (Cognition, Behaviour, Technology) Lab, 1 Institut national de recherche en informatique et en automatique (INRIA), Valbonne, France; ^2^ki:elements GmbH, Saarbrücken, Germany

## Abstract

**Introduction:**

One of the major challenges in clinical psychiatry remains the absence of well established objective measures of symptoms’ severity. Clinical insights are mainly provided through keen behavioral observation and subjective questionnaires and scales.

**Objectives:**

The aim of this paper is to predict depression severity through speech using the features extracted from the speech as provided by participants during a semi-structured dialogue with a virtual avatar.

**Methods:**

We use data from a subset of the DAICWOZ dataset consisting in 142 dialogues between participants and a virtual avatar during which the avatar uses several prompts to maintain a conversation with the participant. The avatar uses prompts involving the topics of travel, dream jobs, and memorable experiences. From the speech generated from the dialogue, we extract participant utterances separated by prompt and extract features from the three sets of transcripts. We extract content features from the transcript and acoustic features from the excerpt corresponding to the speech from the participant for the prompt in question.We perform regression experiments on the PHQ8 items using the features extracted from each set of transcripts. Furthermore, we combine the features extracted from each set of transcripts and compute partial spearman correlations between them and the PHQ8 items using gender as a covariate.

**Results:**

With our best regression model we obtain an R2 of 0.1, explaining 10% of the variance in the PHQ total score. Additionally, we obtain a mean absolute error of 1.25, suggesting that the regressor can detect with more or less precision clinically meaningful differences in depression severity between participants. Partial correlations between the total score and the features show significant correlations between features dependent on the amount of speech generated by each participant, along with the complexity of syntactic structures used.

**Conclusions:**

Automatic analysis of spontaneous speech could help with the detection and monitoring of signs of depression. By combining the use of this technology with timely intervention strategies for instance provided by a virtual agent it could contribute to timely prevention.

**Disclosure of Interest:**

A. König: None Declared, M. Mina Employee of: ki:elements GmbH, S. Schäfer Employee of: ki:elements GmbH, N. Linz Shareolder of: ki:elements GmbH, Employee of: ki:elements GmbH, J. Tröger Shareolder of: ki:elements GmbH, Employee of: ki:elements GmbH

